# Hip fracture in the elderly multidisciplinary rehabilitation (FEMuR) feasibility study: testing the use of routinely collected data for future health economic evaluations

**DOI:** 10.1186/s40814-018-0269-5

**Published:** 2018-05-07

**Authors:** Nefyn H. Williams, Kevin Mawdesley, Jessica L. Roberts, Nafees Ud Din, Nicola Totton, Joanna M. Charles, Zoe Hoare, Rhiannon T. Edwards

**Affiliations:** 1 Betsi Cadwaladr University Health Board, St Asaph, UK; 2 Department of Health Services Research, University of Liverpool, Waterhouse Block B, 1-5 Brownlow Street, Liverpool, L69 3GL UK; 3 Red Wharf Systems, Anglesey, UK; 4 School of Healthcare Sciences, Bangor University, Bangor, UK; 5 School of Health and Related Research, University of Sheffield, Sheffield, UK

**Keywords:** Feasibility study, Economic evaluation, Proximal femoral fracture, Hip fracture, Rehabilitation, Electronic medical record, Health service resource use, Client Service Receipt Inventory, Intraclass correlation coefficient

## Abstract

**Background:**

Health economic evaluations rely on the accurate measurement of health service resource use in order to calculate costs. These are usually measured with patient completed questionnaires using instruments such as the Client Service Receipt Inventory (CSRI). These rely on participants’ recall and can be burdensome to complete. Health service activity data are routinely captured by electronic databases.

The aim was to test methods for obtaining these data and compare with those data collected using the CSRI, within a feasibility study of an enhanced rehabilitation intervention following hip fracture (Fracture in the Elderly Multidisciplinary Rehabilitation: FEMuR).

**Methods:**

Primary care activity including prescribing data was obtained from the Secure Anonymised Information Linkage (SAIL) Databank and secondary care activity (Emergency Department attendances, out-patient visits and in-patient days) directly from Betsi Cadwaladr University Health Board (BCUHB), North Wales, UK. These data were compared with patient responses from the CSRI using descriptive statistics and the intraclass correlation coefficient (ICC).

**Results:**

It was possible to compare health service resource use data for 49 out of 61 participants in the FEMuR study. For emergency department (ED) attendances, records matched in 23 (47%) cases, 21 (43%) over-reported on electronic records compared with CSRI and five participants (10%) under-reported, with an overall ICC of 0.42. For out-patient episodes, records matched in only six cases, 28 participants over-reported on electronic records compared with CSRI and 15 (12%) under-reported, with an overall ICC of only 0.27. For in-patient days, records matched exactly in only five cases (10%), but if an error margin of 7 days was allowed, then agreement rose to 39 (66%) cases, and the overall ICC for all data was 0.88.

It was only possible to compare prescribing data for 12 participants. For prescribing data, the SAIL data reported 117 out of 118 items (99%) and the CSRI only 89 (79%) items.

**Conclusions:**

The use of routinely collected data has the potential to improve the efficiency of trials and other studies. Although the methodology to make the data available has been demonstrated, the data obtained was incomplete and the validity of using this method remains to be demonstrated.

**Trial registration:**

Trial registration: ISRCTN22464643 Registered 21 July 2014.

**Electronic supplementary material:**

The online version of this article (10.1186/s40814-018-0269-5) contains supplementary material, which is available to authorized users.

## Background

Economic evaluations of healthcare and social care interventions are frequently carried out alongside pragmatic randomised controlled trials (RCTs). This involves the measurement of costs and health outcomes, and their comparison either in a disaggregated cost consequences analysis or combined in a cost-minimisation, cost-effectiveness, cost-utility or cost-benefit analysis approach [1]. Costs are typically obtained by measuring health service resource use (or other activity depending upon the perspective of the evaluation) and multiplying this by unit costs obtained from national and local sources [2, 3].

A range of methods have been developed to measure this health service resource use: extracting data from routine primary or secondary care records, patient diaries of resources received, patient self-completed questionnaires and patient interviews [4]. The Client Service Receipt Inventory (CSRI) [5–7] is an example of a patient completed questionnaire, which collects retrospective information about study participants’ use of health services and other services such as social care, voluntary services from charities, etc. It is a bespoke questionnaire developed to gather information on key health and social care contacts based on the perspective of the economic analysis. In the case of health service use, participants are asked about the number of consultations with primary care services, for example general practitioners (GPs), nurses, pharmacists; the number of consultations with community services, for example district nurses, therapists; secondary care out-patient appointments; attendance at the emergency department (ED); in-patient days and procedures and prescribed drugs and dosage. Other examples can be found at the Database of Instruments for Resource Use Measurement (DIRUM) [8]. Proponents of this approach argue that it offers an opportunity to ask participants about their contacts with a wide range of services spanning health care, social care and the voluntary sector. This can be self-completed either by the patient or with the assistance of a researcher. Mistry et al. [4] compared health service use data from patient self-reported questionnaires with data extracted from GP records and found that the level of agreement was moderate and that the recorded number of contacts was higher for patient questionnaires than for GP records. They argued that there was under-reporting of resource use in GP notes. Critics argue that the disadvantage of gathering such resource use data in this way is that it can be time consuming to complete, requires accurate recall by participants, which is particularly difficult for those with cognitive impairment, and can be burdensome [9].

Much of this health service activity data is routinely collected on computerised health records. For example, information on emergency department attendances, hospital admissions and out-patient appointments is collected on patient administration systems; information on general practice consultations and prescribing is collected on computerised record database of general practice. The value of such routinely collected health data is well recognised and there have been a number of strategic initiatives to collect and link data within large datasets for research and other purposes.

The aim of this study was to test methods for obtaining routinely collected data on health service use, evaluate the quality of the data acquired, and compare these data with data collected using the CSRI over the same time period. This would have the potential to reduce participant burden and increase the efficiency of RCT methods in the future.

### The Secure Anonymised Information Linkage Databank in Wales

In Wales, the Secure Anonymised Information Linkage (SAIL) Databank is a repository of personal data records for the population of Wales [10]. Data held therein, including health and social care data for individuals, has been anonymised but the data held for each person from whatever source can be linked and made available for research. Data linkage allows researchers to use existing collections of extensive data that have been routinely collected to address research questions. When adding data to the SAIL Databank, data linkage and anonymisation is achieved by splitting the data sent from each source. Datasets are split into a demographic component (comprising commonly recognised identifiers) and clinical or event component (such as medication records and procedures). The demographic component is transferred to the NHS Wales Informatics Service (NWIS), whilst the clinical component, with no identifying data, goes to the SAIL Databank. NWIS anonymise and encrypt the demographic data, each individual record being assigned an Anonymous Linking Field (ALF) generated from a person’s demographic details. These anonymised demographic elements of the datasets are then sent to SAIL. They contain only the ALF, week of birth, gender code and broad area of residence (divided into blocks of approximately 1500 head of population). The records are then recombined with the clinical component of the dataset. Because the ALF generated for each individual will always be the same, this can be used to link the newly supplied data to other data held by SAIL for each individual, whilst retaining anonymity. The records are thus ready for linkage to other datasets for research use. In 2015, 70% of general medical practices in Wales contributed data to SAIL [11]. At a local level, individual health organisations have also developed their own information warehouses.

## Methods

In order to improve the care of elderly patients who have suffered a proximal femoral hip fracture, commonly known as a hip fracture, an enhanced community-based rehabilitation programme was developed [12]. The methods for a future definitive randomised controlled trial of this enhanced intervention, compared with usual rehabilitation, were tested in a randomised feasibility study [13, 14]. Sixty-one participants were randomised with mean age 79.4 years, 75% were female and 51% lived alone. Types of fracture are as follows: 44% intra-capsular, 33% extra-capsular. Types of surgery are as follows: 8% total hip arthroplasty, 48% hemi-arthroplasty and 31% internal fixation. This included a concurrent economic evaluation, where a researcher at baseline and after a 3-month follow-up administered the CSRI (Additional files 1 and 2).

Health service use data from the patient-completed CSRI were compared with those obtained from routinely collected data on computerised patient records collected over the same time period from two sources: secondary care activity from the Betsi Cadwaladr University Health Board (BCUHB) information warehouse; primary care activity and prescribing from the SAIL databank. Informed patient consent was obtained as part of the FEMuR randomised feasibility study, which included a section on the use of healthcare records that had been approved by NHS Wales Research Ethics Committee 5.

**Fig. 1:**
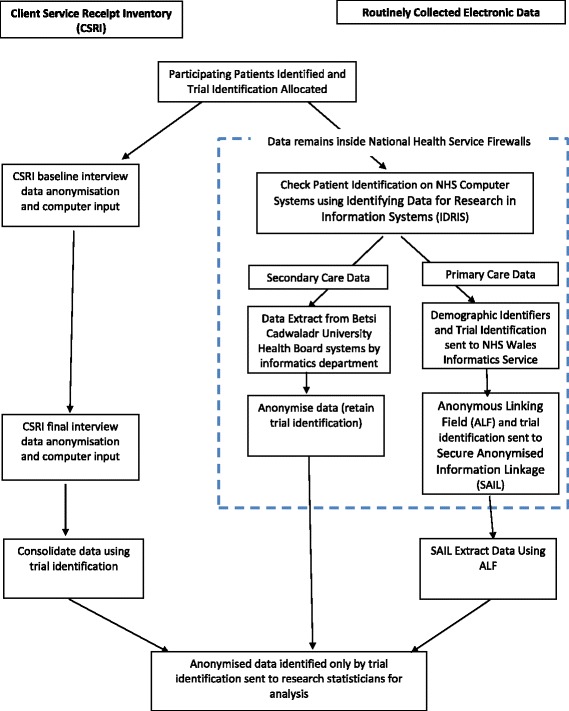
Information flow diagram

In North Wales, the ‘Health Data Platform’ project was instigated as a collaboration between BCUHB and Bangor University. This project sought to circumvent the problems associated with making routinely collect health data available for research, such as ensuring anonymity and data protection, by designing and implementing methods to handle, transfer and store the data (Fig. 1).

### Identifying patients

The next step was to check the identity of the trial participants to ensure that the health records extracted were for the correct person. This check was undertaken through IDRIS (Identifying Data for Research in Information Systems), a bespoke software system designed by the North Wales Organisation for Randomised Trials in Health (NWORTH), the Clinical Trials Unit in Bangor, and developed by the BCUHB informatics department. The identification number, surname, forename, date of birth, gender, postcode and NHS number of each of the trial participants were inputted into IDRIS, which resides on servers within the BCUHB network. Participant information was checked in real time against data held on the Master Patient Index at the Welsh Demographic Service (WDS) to confirm (or fail to confirm) the identity of the participants. If the enquiry to the WDS failed to confirm a participant’s identity, no data for that participant would be made available to the researchers.

### Extracting data

Each patient whose identity was confirmed by IDRIS was added to a register containing their details and participant identification number. Once all participants had been checked by IDRIS, the register was closed and sent electronically to the BCUHB informatics department, remaining on BCUHB secure servers at all times. The following data were then extracted from BCUHB systems: in-patient days and episodes, out-patient episodes and ED episodes. Data were anonymised, and patient-identifiable information was removed, in accordance with BCUHB information governance instructions, leaving only the participant identification number to link the records to the other anonymised data held for each patient. The data were then transferred to the study researchers using a secure file transfer system. The data for participants who withdrew from the trial were removed from the IDRIS register and their data were not transferred.

Identifying demographic data for each participant were sent to NWIS by the FEMuR researchers as described above, together with the FEMuR participation identification for each individual. NWIS then created the ALF and sent this to SAIL, together with the participation identifications. SAIL were then able to link the ALFs to data held in SAIL for each individual and send the data to the research team for analysis. The following data were extracted from the SAIL databank: primary care consultations, and prescribed medication.

### Comparing data

Data concerning ED attendances, out-patient episodes and in-patient days from BCUHB systems were compared with data obtained from the CSRI questionnaire for consistency using the intraclass correlation coefficient (ICC) [15]. The number of contacts recorded for both data collection methods, their resulting ICC and corresponding *p* values to indicate statistical significance have been presented in Table 1. Values less than 0.5, between 0.5 and 0.75, between 0.75 and 0.9, and greater than 0.9 indicate poor, moderate, good and excellent reliability respectively [16]. Data concerning prescribing were obtained from the SAIL databank and compared using descriptive statistics.

**Table 1:** Comparison of health service activity data from electronic records compared with data from the Client Service Receipt Inventory

	Number of episodes recorded		Compared with Client Service Receipt Inventory (*n* = 49)				
	Electronic record	Client Service Receipt Inventory	Matched	Under-reported	Over-reported	ICC (95% CI)	Test statistic of ICC F_(48,48)_ *p* value
Emergency department attendances	60	43	23 (47%)	5 (10%)	21 (43%)	0.42 (0.16 to 0.63)	2.45, *p* < 0.001
Out-patient episodes	215	204	6 (12%)	15 (31%)	28 (57%)	0.27 (−0.006 to 0.51)	1.75, *p* = 0.028
In-patient days	1274	1247	5 (10%)	24 (49%)	20 (41%)	0.88 (0.79 to 0.93)	15.31, *p* < 0.001

## Results

### Ability to define patients from routine data: patient details in IDRIS

Personal details for each of the 61 patients who consented to join the FEMuR trial were input into IDRIS. IDRIS requires a perfect match for surname, date of birth, gender and postcode for the identification check to be positive and this was immediately obtained for 41 patients. Of those that failed the identification check, the majority were because of incorrect postcodes. Discrepancies were occurring because the trial records held the primary address of the patient but hospital records held the postcode of the address to which the patient had been discharged, which was often the residence of a carer or close relative. When these postcodes were corrected, there remained three patients without positive identification. This occurred because some types of update of hospital records temporarily left multiple records on the Master Patient Index for a number of days, until the records were merged into one. The rules by which IDRIS was developed preclude positive checks when multiple records were returned and there was no mechanism to override this, so these three patients had to be excluded. One participant did not complete the CSRI at follow-up, and participant withdrawal further reduced the number of patients in the triangulation study to 49.

### Data validation

The CSRI data were subject to data validation within the FEMuR trial process (as defined in the trial’s data management plan). During this validation, three data input errors were identified, when transcribing from source paper data to the electronic database. The errors found were corrected, but as they were within the pre-specified 2% error rate to trigger a full data entry audit, no further action was required.

### Emergency department attendances

In total, 60 ED attendances were logged in electronic medical records compared with 43 reported in the CSRI data (Table 1). Records from both sources matched in 23 of the 49 (47%) participants, 21 (43%) over-reported attendance on BCUHB records compared with CSRI and five (10%) under-reported attendance. Two participants had ED episodes shortly before the reported data range and these might have been reported in error. Other possible causes of these discrepancies were loss of NHS records or episodes in which the patient presented directly to the acute medical unit, which was mistaken for the ED. The single-measure ICC was 0.42 (95% CI 0.16 to 0.63) which suggests poor agreement.

### Outpatient episodes

The overall over-reporting of out-patient episodes in the electronic medical record data compared with the CSRI was less pronounced, with 215 reported episodes in the electronic medical records compared with 204 reported in the CSRI (Table 1). Records from both sources matched for only six participants (12%), with 28 participants (57%) over-reporting and 15 participants (31%) under-reporting compared with the medical records. The ICC was 0.27 (95% CI − 0.006 to 0.51) suggesting that there was little similarity between the two data collection methods. Further examination of the CSRI data for two participants who were outliers (one participant reported 30 in the CSRI data, but only five in the medical records; the other patient reported 42 in the CSRI data, with three logged in the medical records) suggested that the CSRI data represented the length in minutes of each episode, rather than the number of episodes. Even accounting for these differences, there was still over-reporting in the electronic medical record compared with the CSRI.

### Inpatient days

The data from the two sources were well matched for in-patient days, with 1247 days recorded on the CSRI forms and 1274 days from electronic medical records. The single-measure ICC was 0.88 (95% CI 0.79 to 0.93), suggesting good agreement. Only five (10%) of the individual patient records matched exactly between the two data sets, which may be because days were a more difficult unit to record accurately than episodes and are more difficult to recall accurately during interview. To illustrate, if an error margin of ± 7 days were allowed, then the data agreed for 41 participants (84%).

### General practice consultations

It was not possible to interpret the GP consultation data using the coding provided. It was not possible to determine whether the GP contacts referred to face-to-face consultations, telephone contacts, result recording, medicine management activities or other administrative tasks. Because of this, it was not possible to compare the number of GP consultations.

### Prescribing data

Only 18 of the 49 participants were matched to data from SAIL. The remainder were from practices that had not consented for their data to be included in the SAIL database. Of the 18 matches, six were discarded because the only data from SAIL were many months prior to the FEMuR study. Table 2 details the matches between CSRI and SAIL for the remaining 12 participants. The 12 participants were prescribed 118 drugs according to either data source in the 3-month period, 88 prescriptions were reported in the CSRI questionnaires and SAIL reported 103 prescriptions in the 3-month period prior to the CSRI interview. However, of the five participants whose CSRI data did not appear in the SAIL databank in its entirety, four had confused dates. The CSRI data for these four were all found in the SAIL data if the 3-month period was extended by 1 day, 5 days, 4 weeks and 4 weeks respectively. Allowing for these date extensions, 117 of the 118 prescriptions (99.2%) were reported on SAIL, compared with 89 (79.4%) in CSRI. The only drug use reported on CSRI that did not appear in SAIL was an unspecified ‘painkiller’ recorded by one participant, which might have been purchased over the counter. More prescription data were obtained from SAIL than from the CSRI; 88 items (75%) matched, but there was an additional 15 items (13%) recorded on SAIL, and a further 14 items (12%) if the SAIL data period were extended to 4 months. It was highly likely that items prescribed immediately before the 3-month period were taken by the participant during this period and recorded on the CSRI.

**Table 2:** Comparison of prescription data obtained from the Client Service Receipt Inventory (CSRI) with that obtained from the Secure Anonymised Information Linkage (SAIL) Database

Study ID	No. of CSRI drugs	No. not matched on SAIL < 3 months	No. not matched on SAIL < 4 months	SAIL drugs not on CSRI	Comment/drugs not matched
1109	9	0	0	3	Lactulose, Portex, Diprobase
1121	12	0	0	4	Naproxen, Voltarol, Diazepam, Fluoxetine
2101	5	1	1	1	Painkiller reported on CSRI and not SAIL (Over The Counter?); Flu vaccine on SAIL but not CSRI
2106	3	0	0	3	Alendronic acid, Adcal, Laxido Orange
2114	9	0	0	1	Ferrous Fumarate
2116	6	0	0	3	Calcium+Cholecalciferol, Alendronic Acid, Sudocrem
2117	7	1	0	0	Missing CSRI matched at 3 months + 1 day
2118	10	0	0	6	Zapain, Novomix, Timodine, Loperamide, Cyclizine, Novotwist
2119	9	4	0	3	Missing CSRI matched at 3 months + 4 weeks; Tramadol, Viscotears, Lacri-Lube on SAIL but not CSRI
2121	2	0	0	4	Tramadol, Paracetamol, Ibuprofen, Diprobase
3108	8	5	0	0	Missing CSRI matched at 3 months + 5 days
3114	8	4	0	1	Missing CSRI matched at 3 months + 4 weeks; Gastrocote on SAIL but not CSRI

## Discussion

### Summary of main findings

It was possible to obtain routinely collected data for comparison with that collected in the CSRI. However, much of this data was incomplete, particularly primary care activity and prescribing from the SAIL database, whose coverage of general medical practices treating patients in the FEMuR study was incomplete. Although some areas such as the number of in-patient days and prescribing showed high levels of comparability between data obtained from medical records and data obtained from the CSRI; there were lower levels of comparability for out-patient appointments and emergency department attendances.

The over-reporting of prescribing data in the electronic medical records may be because some participants forgot about some of their prescribed items, or considered them unimportant or irrelevant, or because they did not collect their prescriptions.

### Strengths and limitations

Provision of routinely collected data using electronic methods for research purposes holds considerable potential for future studies. In this study, a novel method was used, which meant that the process of obtaining the data from hospital records available was slow and laborious. The data required were confidential and of a potentially sensitive nature, and each stage of the process required careful planning and liaison with stakeholders to ensure that the correct procedures and security processes were followed. In particular, liaison with the data keepers and BCUHB information governance had to progress with care and there were understandable and valid delays. Despite these challenges, the data were successfully obtained and the software and methodology are now in place. Future uses of this process would be much more streamlined whilst still ensuring that security procedures and good practice were adhered to.

Although it was possible to account for some of the discrepancies in the data by identifying data input errors or accounting for errors in patient recall, it was not possible to check the accuracy of the data collected from BCUHB systems. This is a current limitation that should be addressed in future work as it is not currently possible to confidently conclude which of these data sets provides the most accurate representation of service use. It was not possible to use any routinely collected data on the number of GP consultations because this was not reliably recorded by the general practice record database.

A further limitation is that currently there is no central database for social care data. Databases are focused on health care and health-related service use, leaving a potential gap in service use reporting if only routinely collected data is gathered. The lack of availability for systems to gather social care service use means that instruments such as the CSRI still need to be administered, whilst these routine systems are developed, especially if social care services are a key consideration for the analysis given the chosen perspective.

### Comparison with previous literature

There have been many studies where routinely collected administration data were compared to data obtained from patient self-reporting but in different circumstances, often in different countries (thus reflecting different methods of collecting routine data and different information technology systems for storage). The results reported have been contradictory and inconclusive. For example, one study reported a high concordance between self-reported and claims-based hospital episodes, but concordance for physician visits was low [17]. Factors significantly associated with bidirectional (over- and under-reporting) and unidirectional (over- or under-reporting) error patterns were detected. Therefore, caution was advised when drawing conclusions based on just one physician visit data source. Consistent with our findings, another study found that patients tended to report less use of physicians than was recorded in the computerised provider records [18]. Survey estimates based on self-report tended to underestimate true health care use in the older population [19]. Depending on the type of service, measure of service use and costs, agreement ranged from excellent to poor and varied substantially between individuals [20]. The different data sources resulted in similar estimates on the population level; however, there were pronounced differences for out-patient visits on an individual level [21]. The accuracy of the results was heavily dependent upon context. For example, GP records provided more accurate data on the use of primary care contacts than patient report, but less-reliable information on contacts with other health services. Thus, reliance on GP records for data on hospital services and other community health services based outside of general practice surgeries was not recommended [22]. A recently published review of studies on the Database of Instruments for Resource-Use Measurement (DIRUM) [8] found evidence for a good correlation between medical records and patient or carer recall [23, 24], but overall the conclusions of Williams et al. [25] remain valid:

- Routine data have the potential to measure patient outcomes and support health technology assessment by RCTs;
- The cost of data collection and analysis is likely to reduce;
- Further work is required to improve the detail, precision and validity of routine data;
- Better knowledge of the capability of local systems and access to the data is needed.

Ridyard and Hughes [23] state there are no universally recognised methods for service use data collection, each method has its advantages and disadvantages. Johnson et al. [26] also state that there is no gold standard for service use measurement, and cite previous studies comparing patient self-report methods with health records that reported substantial agreement between patient self-report and medical records [27–29]. The findings of this study showed that there was agreement between the two methods for in-patient data and prescribing, but less agreement for emergency episodes of care and out-patient appointments. It should be noted that the different approaches may lead to over- or underestimation of costs in further analyses such as cost-effectiveness analysis. However, with no gold standards of service use data collection [23, 24], trials need to ensure that they collect the service use data relevant to the population and intervention under investigation, using the most appropriate methods. This is also recommended by The International Society for Pharmacoeconomics and Outcomes Research (ISPOR) RCT Cost-Effectiveness Analysis (CEA) Task Force Report [30]. Health economists need to be clear on their perspective, data collection methods and choice of services to be collected, so that it is clear to the reader how the analysis was conducted and why certain methods were used, in order for the results to be fully understood.

## Conclusions

The use of routinely collected data has the potential to improve the efficiency of trials and other studies. Although the methodology to make the data available has been demonstrated, the data obtained were incomplete and the validity of using this method remains to be demonstrated. Further investigation is required to evaluate the quality and accuracy of the data and to test the validity of different data sources in different contexts. In the meantime, health economic analysis should collect service use data relevant to the population and intervention under investigation, using the most appropriate methods [30]. These include extracting data from routine primary or secondary care records, patient diaries of resources received, patient self-completed questionnaires, patient interviews or a mixture of methods.

## Additional files

Additional file 1: Fracture in the Elderly Multidisciplinary Rehabilitation (FEMuR) baseline Client Service Receipt Inventory (CSRI) questionnaire. (DOCX 35 kb)

Additional file 2: Fracture in the Elderly Multidisciplinary Rehabilitation (FEMuR) 3-month follow-up Client Service Receipt Inventory (CSRI) questionnaire. (DOCX 72 kb)

## Abbreviations

ALF: Anonymous Linking Field
BCUHB: Betsi Cadwaladr University Health Board
CEA: Cost-effectiveness analysis
CSRI: Client Service Receipt Inventory
DIRUM: Database of Instruments for Resource Use Measurement
ED: Emergency department
FEMuR: Fracture in the Elderly Multidisciplinary Rehabilitation
GP: General practitioner
ICC: Intraclass correlation coefficient
IDRIS: Identifying Data for Research in Information Systems
ISPOR: International Society for Pharmacoeconomics and Outcomes Research
NHS: National Health Service
NWIS: NHS Wales Informatics Service
NWORTH: North Wales Organisation for Randomised Trials in Health
RCT: Randomised controlled trial
SAIL: Secure Anonymised Information Linkage
WDS: Welsh Demographic Service
