# Supplementary material for: Hip fracture in the elderly multidisciplinary rehabilitation (FEMuR) feasibility study: testing the use of routinely collected data for future health economic evaluations
Source: Pilot Feasibility Stud. 2018 May 7;4:76. doi: 10.1186/s40814-018-0269-5 (PMC5937043; doi:10.1186/s40814-018-0269-5)
Supplement: Supplementary file 1 — Fracture in the Elderly Multidisciplinary Rehabilitation (FEMuR) baseline Client Service Receipt Inventory (CSRI) questionnaire. (DOCX 35 kb) [file 40814_2018_269_MOESM1_ESM.docx]

| **1.1 Hospital Service Use** |
| --- |
| **Interviewer instructions: Please complete the table to show the hospital services that the participant has used over the last 3 months.** |

**FEMuR: Fracture in the Elderly Multidisciplinary Rehabilitation Baseline Service Use Questionnaire**

| **Service used by participant** | **Name of ward, clinic, hospital or centre (including acute or community hospital)** | **Reason for using service (e.g. nature of illness)** | **Unit of measurement** | **Total number of units received** | | | | **Was this a Readmission?**  **Yes/No** | | **Was this hip related?**  **Yes/No** |
| --- | --- | --- | --- | --- | --- | --- | --- | --- | --- | --- |
| **Inpatient Ward**  1. |  |  | Inpatient day |  |  |  |  |  |  |  |
|  |  |  |  |  |  |  |  |  |  |  |
|  |  |  |  |  |  |  |  |  |  |  |
| 2. |  |  | Inpatient day |  |  |  |  |  |  |  |
|  |  |  |  |  |  |  |  |  |  |  |
|  |  |  |  |  |  |  |  |  |  |  |
| 3. |  |  | Inpatient day |  |  |  |  |  |  |  |
|  |  |  |  |  |  |  |  |  |  |  |
|  |  |  |  |  |  |  |  |  |  |  |
| 4. |  |  | Inpatient day |  |  |  |  |  |  |  |
|  |  |  |  |  |  |  |  |  |  |  |
|  |  |  |  |  |  |  |  |  |  |  |
| **Outpatient Appointment**  1. |  |  | Appointment |  |  |  |  |  |  |  |
|  |  |  |  |  |  |  |  |  |  |  |
|  |  |  |  |  |  |  |  |  |  |  |
| 2. |  |  | Appointment |  |  |  |  |  |  |  |
|  |  |  |  |  |  |  |  |  |  |  |
|  |  |  |  |  |  |  |  |  |  |  |
| 3. |  |  | Appointment |  |  |  |  |  |  |  |
|  |  |  |  |  |  |  |  |  |  |  |
|  |  |  |  |  |  |  |  |  |  |  |
| 4. |  |  | Appointment |  |  |  |  |  |  |  |
|  |  |  |  |  |  |  |  |  |  |  |
|  |  |  |  |  |  |  |  |  |  |  |

| **1.1 Hospital Service Use continued** |
| --- |
| **Interviewer instructions: Please complete the table to show the hospital services that the participant has used over the last 3 months.** |

| **Service used by participant** | **Name of ward, clinic, hospital or centre (including acute or community hospital)** | **Reason for using service (e.g. nature of illness, regular respite arrangement)** | **Unit of measurement** | **Total number of units received** | | | | | **Was this a Readmission?**  **Yes/No** | **Was this hip related?**  **Yes/No** |
| --- | --- | --- | --- | --- | --- | --- | --- | --- | --- | --- |
| **Accident and Emergency**  1. |  |  | Attendance |  |  |  |  |  |  |  |
|  |  |  |  |  |  |  |  |  |  |  |
|  |  |  |  |  |  |  |  |  |  |  |
| 2. |  |  | Attendance |  |  |  |  |  |  |  |
|  |  |  |  |  |  |  |  |  |  |  |
|  |  |  |  |  |  |  |  |  |  |  |
| 3. |  |  | Attendance |  |  |  |  |  |  |  |
|  |  |  |  |  |  |  |  |  |  |  |
|  |  |  |  |  |  |  |  |  |  |  |
| 4. |  |  | Attendance |  |  |  |  |  |  |  |
|  |  |  |  |  |  |  |  |  |  |  |
|  |  |  |  |  |  |  |  |  |  |  |
| **Day Hospital**  1. |  |  | Day Attendance |  |  |  |  |  |  |  |
|  |  |  |  |  |  |  |  |  |  |  |
|  |  |  |  |  |  |  |  |  |  |  |
| 2. |  |  | Day Attendance |  |  |  |  |  |  |  |
|  |  |  |  |  |  |  |  |  |  |  |
|  |  |  |  |  |  |  |  |  |  |  |
| 3. |  |  | Day Attendance |  |  |  |  |  |  |  |
|  |  |  |  |  |  |  |  |  |  |  |
|  |  |  |  |  |  |  |  |  |  |  |
| 4. |  |  | Day Attendance |  |  |  |  |  |  |  |
|  |  |  |  |  |  |  |  |  |  |  |
|  |  |  |  |  |  |  |  |  |  |  |
| **Other Hospital Services**  1. |  |  | Please specify: |  |  |  |  |  |  |  |
|  |  |  |  |  |  |  |  |  |  |  |
|  |  |  |  |  |  |  |  |  |  |  |
| 2. |  |  | Please specify: |  |  |  |  |  |  |  |
|  |  |  |  |  |  |  |  |  |  |  |
|  |  |  |  |  |  |  |  |  |  |  |
| 3. |  |  | Please specify: |  |  |  |  |  |  |  |
|  |  |  |  |  |  |  |  |  |  |  |
|  |  |  |  |  |  |  |  |  |  |  |
| 4. |  |  | Please specify: |  |  |  |  |  |  |  |
|  |  |  |  |  |  |  |  |  |  |  |
|  |  |  |  |  |  |  |  |  |  |  |

| **This section asks about the health and social care services that you have used over the past 3 months.** |
| --- |
| **It also asks about the medications that you use.** |

| **1.2 Community Based Service Use** |
| --- |
| **Interviewer instructions: Please complete the table to show the community based services that the participant has used over the last 3 months.** |
| ***Please do not include services provided by people employed directly by the accommodation facility in which the participant was living at the time.*** |

| **Service used by participant** | **Number of home visits** | | | | **Number of visits to surgery or clinic** | | | | **Provider agency (please tick)** | | | | | | | | | | | | **Average duration**  **of contact (minutes)** | | | | | **Was this hip related?**  **Yes/No** |
| --- | --- | --- | --- | --- | --- | --- | --- | --- | --- | --- | --- | --- | --- | --- | --- | --- | --- | --- | --- | --- | --- | --- | --- | --- | --- | --- |
|  |  |  |  |  |  |  |  |  | **NHS** | | | **Local authority** | | | **Voluntary organisation** | | | **Private organisation** | | |  |  |  |  |  |  |
|  |  |  |  |  |  |  |  |  |  |  |  |  |  |  |  |  |  |  |  |  |  |  |  |  |  |  |
| Physiotherapist |  |  |  |  |  |  |  |  |  |  |  |  |  |  |  |  |  |  |  |  |  |  |  |  |  |  |
|  |  |  |  |  |  |  |  |  |  |  |  |  |  |  |  |  |  |  |  |  |  |  |  |  |  |  |
|  |  |  |  |  |  |  |  |  |  |  |  |  |  |  |  |  |  |  |  |  |  |  |  |  |  |  |
| Occupational health therapist |  |  |  |  |  |  |  |  |  |  |  |  |  |  |  |  |  |  |  |  |  |  |  |  |  |  |
|  |  |  |  |  |  |  |  |  |  |  |  |  |  |  |  |  |  |  |  |  |  |  |  |  |  |  |
|  |  |  |  |  |  |  |  |  |  |  |  |  |  |  |  |  |  |  |  |  |  |  |  |  |  |  |
|  |  |  |  |  |  |  |  |  |  |  |  |  |  |  |  |  |  |  |  |  |  |  |  |  |  |  |
| General practitioner |  |  |  |  |  |  |  |  |  |  |  |  |  |  |  |  |  |  |  |  |  |  |  |  |  |  |
|  |  |  |  |  |  |  |  |  |  |  |  |  |  |  |  |  |  |  |  |  |  |  |  |  |  |  |
|  |  |  |  |  |  |  |  |  |  |  |  |  |  |  |  |  |  |  |  |  |  |  |  |  |  |  |
| Practice nurse (GP clinic) |  |  |  |  |  |  |  |  |  |  |  |  |  |  |  |  |  |  |  |  |  |  |  |  |  |  |
|  |  |  |  |  |  |  |  |  |  |  |  |  |  |  |  |  |  |  |  |  |  |  |  |  |  |  |
|  |  |  |  |  |  |  |  |  |  |  |  |  |  |  |  |  |  |  |  |  |  |  |  |  |  |  |
|  |  |  |  |  |  |  |  |  |  |  |  |  |  |  |  |  |  |  |  |  |  |  |  |  |  |  |
