# Supplementary material for: Hip fracture in the elderly multidisciplinary rehabilitation (FEMuR) feasibility study: testing the use of routinely collected data for future health economic evaluations
Source: Pilot Feasibility Stud. 2018 May 7;4:76. doi: 10.1186/s40814-018-0269-5 (PMC5937043; doi:10.1186/s40814-018-0269-5)
Supplement: Supplementary file 2 — Fracture in the Elderly Multidisciplinary Rehabilitation (FEMuR) 3-month follow-up Client Service Receipt Inventory (CSRI) questionnaire. (DOCX 72 kb) [file 40814_2018_269_MOESM2_ESM.docx]

**Fracture in the Elderly Multidisciplinary Rehabilitation (FEMuR)** **3 Month Follow-up Service Use Questionnaire**

**1.1 Hospital Service Use
Interviewer instructions: Please complete the table to show the hospital services that the participant has used over the last 3 months.**

| **Service used by participant** | **Name of ward, clinic, hospital or centre (including acute or community hospital)** | **Reason for using service (e.g. nature of illness)** | **Unit of measurement** | **Total number of units received** | | | | **Was this a Readmission?**  **Yes/No** | | **Was this hip related?**  **Yes/No** |
| --- | --- | --- | --- | --- | --- | --- | --- | --- | --- | --- |
| **Orthopaedic Trauma Inpatient Ward immediately following hip fracture** |  |  | Inpatient day |  |  |  |  |  |  |  |
|  |  |  |  |  |  |  |  |  |  |  |
|  |  |  |  |  |  |  |  |  |  |  |
| **Surgical Inpatient Ward immediately following hip fracture** |  |  | Inpatient day |  |  |  |  |  |  |  |
|  |  |  |  |  |  |  |  |  |  |  |
|  |  |  |  |  |  |  |  |  |  |  |
| **Assessment Inpatient Ward**  1. |  |  | Inpatient day |  |  |  |  |  |  |  |
|  |  |  |  |  |  |  |  |  |  |  |
|  |  |  |  |  |  |  |  |  |  |  |
| 2. |  |  | Inpatient day |  |  |  |  |  |  |  |
|  |  |  |  |  |  |  |  |  |  |  |
|  |  |  |  |  |  |  |  |  |  |  |
| 3. |  |  | Inpatient day |  |  |  |  |  |  |  |
|  |  |  |  |  |  |  |  |  |  |  |
|  |  |  |  |  |  |  |  |  |  |  |
| **Rehabilitation Inpatient Ward**  1. |  |  | Inpatient day |  |  |  |  |  |  |  |
|  |  |  |  |  |  |  |  |  |  |  |
|  |  |  |  |  |  |  |  |  |  |  |
| 2. |  |  | Inpatient day |  |  |  |  |  |  |  |
|  |  |  |  |  |  |  |  |  |  |  |
|  |  |  |  |  |  |  |  |  |  |  |
| 3. |  |  | Inpatient day |  |  |  |  |  |  |  |
|  |  |  |  |  |  |  |  |  |  |  |
|  |  |  |  |  |  |  |  |  |  |  |
| **Assessment Outpatient Appointment**  1. |  |  | Appointment |  |  |  |  |  |  |  |
|  |  |  |  |  |  |  |  |  |  |  |
|  |  |  |  |  |  |  |  |  |  |  |
| 2. |  |  | Appointment |  |  |  |  |  |  |  |
|  |  |  |  |  |  |  |  |  |  |  |
|  |  |  |  |  |  |  |  |  |  |  |
| 3. |  |  | Appointment |  |  |  |  |  |  |  |
|  |  |  |  |  |  |  |  |  |  |  |
|  |  |  |  |  |  |  |  |  |  |  |
| **Service used by participant** | **Name of ward, clinic, hospital or centre (including acute or community hospital)** | **Reason for using service (e.g. nature of illness)** | **Unit of measurement** | **Total number of units received** | | | | | **Was this a Readmission?**  **Yes/No** | **Was this hip related?**  **Yes/No** |
| **Rehabilitation Outpatient Appointment**  1. |  |  | Appointment |  |  |  |  |  |  |  |
|  |  |  |  |  |  |  |  |  |  |  |
|  |  |  |  |  |  |  |  |  |  |  |
| 2. |  |  | Appointment |  |  |  |  |  |  |  |
|  |  |  |  |  |  |  |  |  |  |  |
|  |  |  |  |  |  |  |  |  |  |  |
| 3. |  |  | Appointment |  |  |  |  |  |  |  |
|  |  |  |  |  |  |  |  |  |  |  |
|  |  |  |  |  |  |  |  |  |  |  |
| **Continuing Care/Respite Inpatient Ward**  1. |  |  | Inpatient day |  |  |  |  |  |  |  |
|  |  |  |  |  |  |  |  |  |  |  |
|  |  |  |  |  |  |  |  |  |  |  |
| 2. |  |  | Inpatient day |  |  |  |  |  |  |  |
|  |  |  |  |  |  |  |  |  |  |  |
|  |  |  |  |  |  |  |  |  |  |  |
| 3. |  |  | Inpatient day |  |  |  |  |  |  |  |
|  |  |  |  |  |  |  |  |  |  |  |
|  |  |  |  |  |  |  |  |  |  |  |
| **Medical Inpatient Ward**  1. |  |  | Inpatient day |  |  |  |  |  |  |  |
|  |  |  |  |  |  |  |  |  |  |  |
|  |  |  |  |  |  |  |  |  |  |  |
| 2. |  |  | Inpatient day |  |  |  |  |  |  |  |
|  |  |  |  |  |  |  |  |  |  |  |
|  |  |  |  |  |  |  |  |  |  |  |
| 3. |  |  | Inpatient day |  |  |  |  |  |  |  |
|  |  |  |  |  |  |  |  |  |  |  |
|  |  |  |  |  |  |  |  |  |  |  |
| **Other Inpatient Ward Services**  1. |  |  | Inpatient day |  |  |  |  |  |  |  |
|  |  |  |  |  |  |  |  |  |  |  |
|  |  |  |  |  |  |  |  |  |  |  |
| 2. |  |  | Inpatient day |  |  |  |  |  |  |  |
|  |  |  |  |  |  |  |  |  |  |  |
|  |  |  |  |  |  |  |  |  |  |  |
| 3. |  |  | Inpatient day |  |  |  |  |  |  |  |
|  |  |  |  |  |  |  |  |  |  |  |
|  |  |  |  |  |  |  |  |  |  |  |
| **Service used by participant** | **Name of ward, clinic, hospital or centre (including acute or community hospital)** | **Reason for using service (e.g. nature of illness, regular respite arrangement)** | **Unit of measurement** | **Total number of units received** | | | | | **Was this a Readmission?**  **Yes/No** | **Was this hip related?**  **Yes/No** |
| **Other Outpatient Services**  1. |  |  | Appointment |  |  |  |  |  |  |  |
|  |  |  |  |  |  |  |  |  |  |  |
|  |  |  |  |  |  |  |  |  |  |  |
| 2. |  |  | Appointment |  |  |  |  |  |  |  |
|  |  |  |  |  |  |  |  |  |  |  |
|  |  |  |  |  |  |  |  |  |  |  |
| 3. |  |  | Appointment |  |  |  |  |  |  |  |
|  |  |  |  |  |  |  |  |  |  |  |
|  |  |  |  |  |  |  |  |  |  |  |
| **Accident and Emergency**  1. |  |  | Attendance |  |  |  |  |  |  |  |
|  |  |  |  |  |  |  |  |  |  |  |
|  |  |  |  |  |  |  |  |  |  |  |
| 2. |  |  | Attendance |  |  |  |  |  |  |  |
|  |  |  |  |  |  |  |  |  |  |  |
|  |  |  |  |  |  |  |  |  |  |  |
| 3. |  |  | Attendance |  |  |  |  |  |  |  |
|  |  |  |  |  |  |  |  |  |  |  |
|  |  |  |  |  |  |  |  |  |  |  |
| **Day Hospital**  1. |  |  | Day Attendance |  |  |  |  |  |  |  |
|  |  |  |  |  |  |  |  |  |  |  |
|  |  |  |  |  |  |  |  |  |  |  |
| 2. |  |  | Day Attendance |  |  |  |  |  |  |  |
|  |  |  |  |  |  |  |  |  |  |  |
|  |  |  |  |  |  |  |  |  |  |  |
| 3. |  |  | Day Attendance |  |  |  |  |  |  |  |
|  |  |  |  |  |  |  |  |  |  |  |
|  |  |  |  |  |  |  |  |  |  |  |
| **Other Hospital Services**  1. |  |  | Please specify: |  |  |  |  |  |  |  |
|  |  |  |  |  |  |  |  |  |  |  |
|  |  |  |  |  |  |  |  |  |  |  |
| 2. |  |  | Please specify: |  |  |  |  |  |  |  |
|  |  |  |  |  |  |  |  |  |  |  |
|  |  |  |  |  |  |  |  |  |  |  |
| 3. |  |  | Please specify: |  |  |  |  |  |  |  |
|  |  |  |  |  |  |  |  |  |  |  |
|  |  |  |  |  |  |  |  |  |  |  |

| **Service used by participant** | **Number of home visits** | | | | **Number of visits to surgery or clinic** | | | | **Provider agency (please tick)** | | | | | | | | | | | | **Average duration**  **of contact (minutes)** | | | | | **Was this hip related?**  **Yes/No** |
| --- | --- | --- | --- | --- | --- | --- | --- | --- | --- | --- | --- | --- | --- | --- | --- | --- | --- | --- | --- | --- | --- | --- | --- | --- | --- | --- |
|  |  |  |  |  |  |  |  |  | **NHS** | | | **Local authority** | | | **Voluntary organisation** | | | **Private organisation** | | |  |  |  |  |  |  |
|  |  |  |  |  |  |  |  |  |  |  |  |  |  |  |  |  |  |  |  |  |  |  |  |  |  |  |
| Physiotherapist |  |  |  |  |  |  |  |  |  |  |  |  |  |  |  |  |  |  |  |  |  |  |  |  |  |  |
|  |  |  |  |  |  |  |  |  |  |  |  |  |  |  |  |  |  |  |  |  |  |  |  |  |  |  |
|  |  |  |  |  |  |  |  |  |  |  |  |  |  |  |  |  |  |  |  |  |  |  |  |  |  |  |
| Occupational health therapist |  |  |  |  |  |  |  |  |  |  |  |  |  |  |  |  |  |  |  |  |  |  |  |  |  |  |
|  |  |  |  |  |  |  |  |  |  |  |  |  |  |  |  |  |  |  |  |  |  |  |  |  |  |  |
|  |  |  |  |  |  |  |  |  |  |  |  |  |  |  |  |  |  |  |  |  |  |  |  |  |  |  |
| Technical Instructors /Rehabilitation Assistants of OTs/Physiotherapists |  |  |  |  |  |  |  |  |  |  |  |  |  |  |  |  |  |  |  |  |  |  |  |  |  |  |
|  |  |  |  |  |  |  |  |  |  |  |  |  |  |  |  |  |  |  |  |  |  |  |  |  |  |  |
|  |  |  |  |  |  |  |  |  |  |  |  |  |  |  |  |  |  |  |  |  |  |  |  |  |  |  |
| District Nurse |  |  |  |  |  |  |  |  |  |  |  |  |  |  |  |  |  |  |  |  |  |  |  |  |  |  |
|  |  |  |  |  |  |  |  |  |  |  |  |  |  |  |  |  |  |  |  |  |  |  |  |  |  |  |
|  |  |  |  |  |  |  |  |  |  |  |  |  |  |  |  |  |  |  |  |  |  |  |  |  |  |  |
| Social Worker |  |  |  |  |  |  |  |  |  |  |  |  |  |  |  |  |  |  |  |  |  |  |  |  |  |  |
|  |  |  |  |  |  |  |  |  |  |  |  |  |  |  |  |  |  |  |  |  |  |  |  |  |  |  |
|  |  |  |  |  |  |  |  |  |  |  |  |  |  |  |  |  |  |  |  |  |  |  |  |  |  |  |
| Community Psychiatric Nurse / Community Mental Health Nurse |  |  |  |  |  |  |  |  |  |  |  |  |  |  |  |  |  |  |  |  |  |  |  |  |  |  |
|  |  |  |  |  |  |  |  |  |  |  |  |  |  |  |  |  |  |  |  |  |  |  |  |  |  |  |
|  |  |  |  |  |  |  |  |  |  |  |  |  |  |  |  |  |  |  |  |  |  |  |  |  |  |  |
| General practitioner |  |  |  |  |  |  |  |  |  |  |  |  |  |  |  |  |  |  |  |  |  |  |  |  |  |  |
|  |  |  |  |  |  |  |  |  |  |  |  |  |  |  |  |  |  |  |  |  |  |  |  |  |  |  |
|  |  |  |  |  |  |  |  |  |  |  |  |  |  |  |  |  |  |  |  |  |  |  |  |  |  |  |
| Practice nurse (GP clinic) |  |  |  |  |  |  |  |  |  |  |  |  |  |  |  |  |  |  |  |  |  |  |  |  |  |  |
|  |  |  |  |  |  |  |  |  |  |  |  |  |  |  |  |  |  |  |  |  |  |  |  |  |  |  |
|  |  |  |  |  |  |  |  |  |  |  |  |  |  |  |  |  |  |  |  |  |  |  |  |  |  |  |
|  |  |  |  |  |  |  |  |  |  |  |  |  |  |  |  |  |  |  |  |  |  |  |  |  |  |  |
|  |  |  |  |  |  |  |  |  |  |  |  |  |  |  |  |  |  |  |  |  |  |  |  |  |  |  |
| Health Visitor |  |  |  |  |  |  |  |  |  |  |  |  |  |  |  |  |  |  |  |  |  |  |  |  |  |  |
|  |  |  |  |  |  |  |  |  |  |  |  |  |  |  |  |  |  |  |  |  |  |  |  |  |  |  |
|  |  |  |  |  |  |  |  |  |  |  |  |  |  |  |  |  |  |  |  |  |  |  |  |  |  |  |
| Psychologist |  |  |  |  |  |  |  |  |  |  |  |  |  |  |  |  |  |  |  |  |  |  |  |  |  |  |
|  |  |  |  |  |  |  |  |  |  |  |  |  |  |  |  |  |  |  |  |  |  |  |  |  |  |  |
|  |  |  |  |  |  |  |  |  |  |  |  |  |  |  |  |  |  |  |  |  |  |  |  |  |  |  |
| Community psychiatrist |  |  |  |  |  |  |  |  |  |  |  |  |  |  |  |  |  |  |  |  |  |  |  |  |  |  |
|  |  |  |  |  |  |  |  |  |  |  |  |  |  |  |  |  |  |  |  |  |  |  |  |  |  |  |
|  |  |  |  |  |  |  |  |  |  |  |  |  |  |  |  |  |  |  |  |  |  |  |  |  |  |  |
| Counsellor |  |  |  |  |  |  |  |  |  |  |  |  |  |  |  |  |  |  |  |  |  |  |  |  |  |  |
|  |  |  |  |  |  |  |  |  |  |  |  |  |  |  |  |  |  |  |  |  |  |  |  |  |  |  |

| **This section asks about the health and social care services that you have used over the past 3 months.** |
| --- |
| **It also asks about the medications that you use.** |

| **1.2 Community Based Service Use** |
| --- |
| **Interviewer instructions: Please complete the table to show the community based services that the participant has used over the last 3 months.** |
| ***Please do not include services provided by people employed directly by the accommodation facility in which the participant was living at the time.*** |

| **Service used by participant** | **Number of home visits** | | | | **Number of visits to surgery or clinic** | | | | **Provider agency (please tick)** | | | | | | | | | | | | **Average duration**  **of contact (minutes)** | | | | | **Was this hip related?**  **Yes/No** |
| --- | --- | --- | --- | --- | --- | --- | --- | --- | --- | --- | --- | --- | --- | --- | --- | --- | --- | --- | --- | --- | --- | --- | --- | --- | --- | --- |
|  |  |  |  |  |  |  |  |  | **NHS** | | | **Local authority** | | | **Voluntary organisation** | | | **Private organisation** | | |  |  |  |  |  |  |
|  |  |  |  |  |  |  |  |  |  |  |  |  |  |  |  |  |  |  |  |  |  |  |  |  |  |  |
| Community Pharmacist |  |  |  |  |  |  |  |  |  |  |  |  |  |  |  |  |  |  |  |  |  |  |  |  |  |  |
|  |  |  |  |  |  |  |  |  |  |  |  |  |  |  |  |  |  |  |  |  |  |  |  |  |  |  |
|  |  |  |  |  |  |  |  |  |  |  |  |  |  |  |  |  |  |  |  |  |  |  |  |  |  |  |
| Home care worker |  |  |  |  |  |  |  |  |  |  |  |  |  |  |  |  |  |  |  |  |  |  |  |  |  |  |
|  |  |  |  |  |  |  |  |  |  |  |  |  |  |  |  |  |  |  |  |  |  |  |  |  |  |  |
|  |  |  |  |  |  |  |  |  |  |  |  |  |  |  |  |  |  |  |  |  |  |  |  |  |  |  |
| Care attendant |  |  |  |  |  |  |  |  |  |  |  |  |  |  |  |  |  |  |  |  |  |  |  |  |  |  |
|  |  |  |  |  |  |  |  |  |  |  |  |  |  |  |  |  |  |  |  |  |  |  |  |  |  |  |

| **1.3 Medication** |
| --- |
| **Interviewer instructions: Please record medications taken by the participant in the last 3 months in the following table. Where possible, please use** |
| **medicine labels and/or prescriptions. If in doubt as to whether to include, please record the details for checking later.** |

| **Medication taken by participant**  **(Tradename)** | **Route of administration**  **(e.g. tablets, injection etc)** | **First Day**  **(dd/mm/yyyy)** | | | | | | | | | | | | **Last Day**  **(dd/mm/yyyy)** | | | | | | | | | | | | **Or**  **Ongoing at date of completing questionnaire (please tick)** | | | **Dose per unit** | **Number of units per day** | | | **Total units prescribed if PRN (as required)** | | | **Was this hip related?**  **Yes/No** |
| --- | --- | --- | --- | --- | --- | --- | --- | --- | --- | --- | --- | --- | --- | --- | --- | --- | --- | --- | --- | --- | --- | --- | --- | --- | --- | --- | --- | --- | --- | --- | --- | --- | --- | --- | --- | --- |
|  |  |  |  |  |  |  |  |  |  |  |  |  |  |  |  |  |  |  |  |  |  |  |  |  |  |  |  |  |  |  |  |  |  |  |  | |
|  |  |  |  |  | / |  |  | / |  |  |  |  |  |  |  |  | / |  |  | / |  |  |  |  |  |  |  |  |  |  |  |  |  |  |  | |
|  |  |  | d | d |  | m | m |  | y | y | y | y |  |  | d | d |  | m | m |  | y | y | y | y |  |  |  |  |  |  |  |  |  |  |  | |
|  |  |  |  |  |  |  |  |  |  |  |  |  |  |  |  |  |  |  |  |  |  |  |  |  |  |  |  |  |  |  |  |  |  |  |  | |
|  |  |  |  |  |  |  |  |  |  |  |  |  |  |  |  |  |  |  |  |  |  |  |  |  |  |  |  |  |  |  |  |  |  |  |  | |
|  |  |  |  |  | / |  |  | / |  |  |  |  |  |  |  |  | / |  |  | / |  |  |  |  |  |  |  |  |  |  |  |  |  |  |  | |
|  |  |  | d | d |  | m | m |  | y | y | y | y |  |  | d | d |  | m | m |  | y | y | y | y |  |  |  |  |  |  |  |  |  |  |  | |
|  |  |  |  |  |  |  |  |  |  |  |  |  |  |  |  |  |  |  |  |  |  |  |  |  |  |  |  |  |  |  |  |  |  |  |  | |
|  |  |  |  |  |  |  |  |  |  |  |  |  |  |  |  |  |  |  |  |  |  |  |  |  |  |  |  |  |  |  |  |  |  |  |  | |
|  |  |  |  |  | / |  |  | / |  |  |  |  |  |  |  |  | / |  |  | / |  |  |  |  |  |  |  |  |  |  |  |  |  |  |  | |
|  |  |  | d | d |  | m | m |  | y | y | y | y |  |  | d | d |  | m | m |  | y | y | y | y |  |  |  |  |  |  |  |  |  |  |  | |
|  |  |  |  |  |  |  |  |  |  |  |  |  |  |  |  |  |  |  |  |  |  |  |  |  |  |  |  |  |  |  |  |  |  |  |  | |
|  |  |  |  |  |  |  |  |  |  |  |  |  |  |  |  |  |  |  |  |  |  |  |  |  |  |  |  |  |  |  |  |  |  |  |  | |
|  |  |  |  |  | / |  |  | / |  |  |  |  |  |  |  |  | / |  |  | / |  |  |  |  |  |  |  |  |  |  |  |  |  |  |  | |
|  |  |  | d | d |  | m | m |  | y | y | y | y |  |  | d | d |  | m | m |  | y | y | y | y |  |  |  |  |  |  |  |  |  |  |  | |
|  |  |  |  |  |  |  |  |  |  |  |  |  |  |  |  |  |  |  |  |  |  |  |  |  |  |  |  |  |  |  |  |  |  |  |  | |
|  |  |  |  |  |  |  |  |  |  |  |  |  |  |  |  |  |  |  |  |  |  |  |  |  |  |  |  |  |  |  |  |  |  |  |  | |
|  |  |  |  |  | / |  |  | / |  |  |  |  |  |  |  |  | / |  |  | / |  |  |  |  |  |  |  |  |  |  |  |  |  |  |  | |
|  |  |  | d | d |  | m | m |  | y | y | y | y |  |  | d | d |  | m | m |  | y | y | y | y |  |  |  |  |  |  |  |  |  |  |  | |
|  |  |  |  |  |  |  |  |  |  |  |  |  |  |  |  |  |  |  |  |  |  |  |  |  |  |  |  |  |  |  |  |  |  |  |  | |
|  |  |  |  |  |  |  |  |  |  |  |  |  |  |  |  |  |  |  |  |  |  |  |  |  |  |  |  |  |  |  |  |  |  |  |  | |
|  |  |  |  |  | / |  |  | / |  |  |  |  |  |  |  |  | / |  |  | / |  |  |  |  |  |  |  |  |  |  |  |  |  |  |  | |
|  |  |  | d | d |  | m | m |  | y | y | y | y |  |  | d | d |  | m | m |  | y | y | y | y |  |  |  |  |  |  |  |  |  |  |  | |
|  |  |  |  |  |  |  |  |  |  |  |  |  |  |  |  |  |  |  |  |  |  |  |  |  |  |  |  |  |  |  |  |  |  |  |  | |
|  |  |  |  |  |  |  |  |  |  |  |  |  |  |  |  |  |  |  |  |  |  |  |  |  |  |  |  |  |  |  |  |  |  |  |  | |
|  |  |  |  |  | / |  |  | / |  |  |  |  |  |  |  |  | / |  |  | / |  |  |  |  |  |  |  |  |  |  |  |  |  |  |  | |
|  |  |  | d | d |  | m | m |  | y | y | y | y |  |  | d | d |  | m | m |  | y | y | y | y |  |  |  |  |  |  |  |  |  |  |  | |
|  |  |  |  |  |  |  |  |  |  |  |  |  |  |  |  |  |  |  |  |  |  |  |  |  |  |  |  |  |  |  |  |  |  |  |  | |
|  |  |  |  |  |  |  |  |  |  |  |  |  |  |  |  |  |  |  |  |  |  |  |  |  |  |  |  |  |  |  |  |  |  |  |  | |
|  |  |  |  |  | / |  |  | / |  |  |  |  |  |  |  |  | / |  |  | / |  |  |  |  |  |  |  |  |  |  |  |  |  |  |  | |
|  |  |  | d | d |  | m | m |  | y | y | y | y |  |  | d | d |  | m | m |  | y | y | y | y |  |  |  |  |  |  |  |  |  |  |  | |
|  |  |  |  |  |  |  |  |  |  |  |  |  |  |  |  |  |  |  |  |  |  |  |  |  |  |  |  |  |  |  |  |  |  |  |  | |
|  |  |  |  |  |  |  |  |  |  |  |  |  |  |  |  |  |  |  |  |  |  |  |  |  |  |  |  |  |  |  |  |  |  |  |  | |
|  |  |  |  |  | / |  |  | / |  |  |  |  |  |  |  |  | / |  |  | / |  |  |  |  |  |  |  |  |  |  |  |  |  |  |  | |
|  |  |  | d | d |  | m | m |  | y | y | y | y |  |  | d | d |  | m | m |  | y | y | y | y |  |  |  |  |  |  |  |  |  |  |  | |
|  |  |  |  |  |  |  |  |  |  |  |  |  |  |  |  |  |  |  |  |  |  |  |  |  |  |  |  |  |  |  |  |  |  |  |  | |
|  |  |  |  |  |  |  |  |  |  |  |  |  |  |  |  |  |  |  |  |  |  |  |  |  |  |  |  |  |  |  |  |  |  |  |  | |
|  |  |  |  |  | / |  |  | / |  |  |  |  |  |  |  |  | / |  |  | / |  |  |  |  |  |  |  |  |  |  |  |  |  |  |  | |
|  |  |  | d | d |  | m | m |  | y | y | y | y |  |  | d | d |  | m | m |  | y | y | y | y |  |  |  |  |  |  |  |  |  |  |  | |
|  |  |  |  |  |  |  |  |  |  |  |  |  |  |  |  |  |  |  |  |  |  |  |  |  |  |  |  |  |  |  |  |  |  |  |  | |

| **Medication taken by participant**  **(Tradename)** | **Route of administration**  **(e.g. tablets, injection etc)** | **First Day**  **(dd/mm/yyyy)** | | | | | | | | | | | | **Last Day**  **(dd/mm/yyyy)** | | | | | | | | | | | | **Or**  **Ongoing at date of completing questionnaire (please tick)** | | | **Dose per unit** | **Number of units per day** | | | **Total units prescribed if PRN (as required)** | | | **Was this hip related?**  **Yes/No** | |
| --- | --- | --- | --- | --- | --- | --- | --- | --- | --- | --- | --- | --- | --- | --- | --- | --- | --- | --- | --- | --- | --- | --- | --- | --- | --- | --- | --- | --- | --- | --- | --- | --- | --- | --- | --- | --- | --- |
|  |  |  |  |  |  |  |  |  |  |  |  |  |  |  |  |  |  |  |  |  |  |  |  |  |  |  |  |  |  |  |  |  |  |  |  | |  |
|  |  |  |  |  |  |  |  |  |  |  |  |  |  |  |  |  |  |  |  |  |  |  |  |  |  |  |  |  |  |  |  |  |  |  |  | |  |
|  |  |  |  |  | / |  |  | / |  |  |  |  |  |  |  |  | / |  |  | / |  |  |  |  |  |  |  |  |  |  |  |  |  |  |  | |  |
|  |  |  | d | d |  | m | m |  | y | y | y | y |  |  | d | d |  | m | m |  | y | y | y | y |  |  |  |  |  |  |  |  |  |  |  | |  |
|  |  |  |  |  |  |  |  |  |  |  |  |  |  |  |  |  |  |  |  |  |  |  |  |  |  |  |  |  |  |  |  |  |  |  |  | |  |
|  |  |  |  |  |  |  |  |  |  |  |  |  |  |  |  |  |  |  |  |  |  |  |  |  |  |  |  |  |  |  |  |  |  |  |  | |  |
|  |  |  |  |  | / |  |  | / |  |  |  |  |  |  |  |  | / |  |  | / |  |  |  |  |  |  |  |  |  |  |  |  |  |  |  | |  |
|  |  |  | d | d |  | m | m |  | y | y | y | y |  |  | d | d |  | m | m |  | y | y | y | y |  |  |  |  |  |  |  |  |  |  |  | |  |
|  |  |  |  |  |  |  |  |  |  |  |  |  |  |  |  |  |  |  |  |  |  |  |  |  |  |  |  |  |  |  |  |  |  |  |  | |  |
|  |  |  |  |  |  |  |  |  |  |  |  |  |  |  |  |  |  |  |  |  |  |  |  |  |  |  |  |  |  |  |  |  |  |  |  | |  |
|  |  |  |  |  | / |  |  | / |  |  |  |  |  |  |  |  | / |  |  | / |  |  |  |  |  |  |  |  |  |  |  |  |  |  |  | |  |
|  |  |  | d | d |  | m | m |  | y | y | y | y |  |  | d | d |  | m | m |  | y | y | y | y |  |  |  |  |  |  |  |  |  |  |  | |  |
|  |  |  |  |  |  |  |  |  |  |  |  |  |  |  |  |  |  |  |  |  |  |  |  |  |  |  |  |  |  |  |  |  |  |  |  | |  |
|  |  |  |  |  |  |  |  |  |  |  |  |  |  |  |  |  |  |  |  |  |  |  |  |  |  |  |  |  |  |  |  |  |  |  |  | |  |
|  |  |  |  |  | / |  |  | / |  |  |  |  |  |  |  |  | / |  |  | / |  |  |  |  |  |  |  |  |  |  |  |  |  |  |  | |  |
|  |  |  | d | d |  | m | m |  | y | y | y | y |  |  | d | d |  | m | m |  | y | y | y | y |  |  |  |  |  |  |  |  |  |  |  | |  |
|  |  |  |  |  |  |  |  |  |  |  |  |  |  |  |  |  |  |  |  |  |  |  |  |  |  |  |  |  |  |  |  |  |  |  |  | |  |
|  |  |  |  |  |  |  |  |  |  |  |  |  |  |  |  |  |  |  |  |  |  |  |  |  |  |  |  |  |  |  |  |  |  |  |  | |  |
|  |  |  |  |  | / |  |  | / |  |  |  |  |  |  |  |  | / |  |  | / |  |  |  |  |  |  |  |  |  |  |  |  |  |  |  | |  |
|  |  |  | d | d |  | m | m |  | y | y | y | y |  |  | d | d |  | m | m |  | y | y | y | y |  |  |  |  |  |  |  |  |  |  |  | |  |
|  |  |  |  |  |  |  |  |  |  |  |  |  |  |  |  |  |  |  |  |  |  |  |  |  |  |  |  |  |  |  |  |  |  |  |  | |  |
|  |  |  |  |  |  |  |  |  |  |  |  |  |  |  |  |  |  |  |  |  |  |  |  |  |  |  |  |  |  |  |  |  |  |  |  | |  |
|  |  |  |  |  | / |  |  | / |  |  |  |  |  |  |  |  | / |  |  | / |  |  |  |  |  |  |  |  |  |  |  |  |  |  |  | |  |
|  |  |  | d | d |  | m | m |  | y | y | y | y |  |  | d | d |  | m | m |  | y | y | y | y |  |  |  |  |  |  |  |  |  |  |  | |  |
|  |  |  |  |  |  |  |  |  |  |  |  |  |  |  |  |  |  |  |  |  |  |  |  |  |  |  |  |  |  |  |  |  |  |  |  | |  |
|  |  |  |  |  |  |  |  |  |  |  |  |  |  |  |  |  |  |  |  |  |  |  |  |  |  |  |  |  |  |  |  |  |  |  |  | |  |
|  |  |  |  |  | / |  |  | / |  |  |  |  |  |  |  |  | / |  |  | / |  |  |  |  |  |  |  |  |  |  |  |  |  |  |  | |  |
|  |  |  | d | d |  | m | m |  | y | y | y | y |  |  | d | d |  | m | m |  | y | y | y | y |  |  |  |  |  |  |  |  |  |  |  | |  |
|  |  |  |  |  |  |  |  |  |  |  |  |  |  |  |  |  |  |  |  |  |  |  |  |  |  |  |  |  |  |  |  |  |  |  |  | |  |
|  |  |  |  |  |  |  |  |  |  |  |  |  |  |  |  |  |  |  |  |  |  |  |  |  |  |  |  |  |  |  |  |  |  |  |  | |  |
|  |  |  |  |  | / |  |  | / |  |  |  |  |  |  |  |  | / |  |  | / |  |  |  |  |  |  |  |  |  |  |  |  |  |  |  | |  |
|  |  |  | d | d |  | m | m |  | y | y | y | y |  |  | d | d |  | m | m |  | y | y | y | y |  |  |  |  |  |  |  |  |  |  |  | |  |
|  |  |  |  |  |  |  |  |  |  |  |  |  |  |  |  |  |  |  |  |  |  |  |  |  |  |  |  |  |  |  |  |  |  |  |  | |  |
|  |  |  |  |  |  |  |  |  |  |  |  |  |  |  |  |  |  |  |  |  |  |  |  |  |  |  |  |  |  |  |  |  |  |  |  | |  |
|  |  |  |  |  | / |  |  | / |  |  |  |  |  |  |  |  | / |  |  | / |  |  |  |  |  |  |  |  |  |  |  |  |  |  |  | |  |
|  |  |  | d | d |  | m | m |  | y | y | y | y |  |  | d | d |  | m | m |  | y | y | y | y |  |  |  |  |  |  |  |  |  |  |  | |  |
|  |  |  |  |  |  |  |  |  |  |  |  |  |  |  |  |  |  |  |  |  |  |  |  |  |  |  |  |  |  |  |  |  |  |  |  | |  |
|  |  |  |  |  |  |  |  |  |  |  |  |  |  |  |  |  |  |  |  |  |  |  |  |  |  |  |  |  |  |  |  |  |  |  |  | |  |
|  |  |  |  |  | / |  |  | / |  |  |  |  |  |  |  |  | / |  |  | / |  |  |  |  |  |  |  |  |  |  |  |  |  |  |  | |  |
|  |  |  | d | d |  | m | m |  | y | y | y | y |  |  | d | d |  | m | m |  | y | y | y | y |  |  |  |  |  |  |  |  |  |  |  | |  |
|  |  |  |  |  |  |  |  |  |  |  |  |  |  |  |  |  |  |  |  |  |  |  |  |  |  |  |  |  |  |  |  |  |  |  |  | |  |

| **1.4 Hip-Fracture Specific Service Use** |
| --- |
| **Interviewer instructions: Please complete the table to show the hip-fracture specific services that the participant has used over the last 3 months.** |
| ***Please do not include services provided by people employed directly by the accommodation facility in which the participant was living at the time.*** |

| **Service used by participant** | **Name of centre/service and location (e.g., outpatient community hospital, inpatient acute hospital)** | **Unit of measurement (e.g., Days, Visits, Classes, etc)** | **Number of units received per week** | | | | | **Total number of units received over the last 3 months** | | | | |
| --- | --- | --- | --- | --- | --- | --- | --- | --- | --- | --- | --- | --- |
|  |  | Please specify: |  |  |  |  |  |  |  |  |  |  |
| Enablement Centre for Rehabilitation Only |  |  |  |  |  |  |  |  |  |  |  |  |
|  |  |  |  |  |  |  |  |  |  |  |  |  |
|  |  | Days |  |  |  |  |  |  |  |  |  |  |
| Nursing Home for Rehabilitation Only |  |  |  |  |  |  |  |  |  |  |  |  |
|  |  |  |  |  |  |  |  |  |  |  |  |  |
|  |  | Classes |  |  |  |  |  |  |  |  |  |  |
| Falls Prevention Classes |  |  |  |  |  |  |  |  |  |  |  |  |
|  |  |  |  |  |  |  |  |  |  |  |  |  |
|  |  | Attendance |  |  |  |  |  |  |  |  |  |  |
| National Exercise Referral Scheme |  |  |  |  |  |  |  |  |  |  |  |  |
|  |  |  |  |  |  |  |  |  |  |  |  |  |
|  |  | Please specify: |  |  |  |  |  |  |  |  |  |  |
| Charity Services (e.g., Red Cross Home from Hospital) |  |  |  |  |  |  |  |  |  |  |  |  |
| 1. |  |  |  |  |  |  |  |  |  |  |  |  |
|  |  | Please specify: |  |  |  |  |  |  |  |  |  |  |
| 2. |  |  |  |  |  |  |  |  |  |  |  |  |
|  |  |  |  |  |  |  |  |  |  |  |  |  |
| 3. |  | Please specify: |  |  |  |  |  |  |  |  |  |  |
|  |  |  |  |  |  |  |  |  |  |  |  |  |
|  |  |  |  |  |  |  |  |  |  |  |  |  |
